# Unveiling the role of histone deacetylases in neurological diseases: focus on epilepsy

**DOI:** 10.1186/s40364-024-00687-6

**Published:** 2024-11-19

**Authors:** Dan-Feng Cao, Xin-Yu Zhou, Qian Guo, Ming-Yao Xiang, Mei-Hua Bao, Bin-Sheng He, Xiao-Yuan Mao

**Affiliations:** 1 Hunan Provincial University Key Laboratory of the Fundamental and Clinical Research on Functional Nucleic Acid, Changsha Medical University, Changsha, 410219 China; 2 Hunan Provincial Key Laboratory of the Research and Development of Novel Pharmaceutical Preparations, Changsha Medical University, Changsha, 410219 China; 3 The First Clinical College, Changsha Medical University, Changsha, 410219 China; 4 Department of Neurosurgery, The Affiliated Hospital of Kangda College of Nanjing Medical University, Lianyungang, 222000 China; 5 Department of Neurology, The Affiliated Lianyungang Hospital of Xuzhou Medical University, Lianyungang, 222000 China; 6 Department of Clinical Pharmacology, Hunan Key Laboratory of Pharmacogenetics and National Clinical Research Center for Geriatric Disorders, Xiangya Hospital, Central South University, Changsha, 410008 China; 7 Institute of Clinical Pharmacology and Engineering Research Center of Applied Technology of Pharmacogenomics of Ministry of Education, Central South University, Changsha, 410078 China

**Keywords:** Epilepsy, Epigenetic targets, Histone deacetylases, Inhibitors, Therapy

## Abstract

Epilepsy remains a prevalent chronic neurological disease that is featured by aberrant, recurrent and hypersynchronous discharge of neurons and poses a great challenge to healthcare systems. Although several therapeutic interventions are successfully utilized for treating epilepsy, they can merely provide symptom relief but cannot exert disease-modifying effect. Therefore, it is of urgent need to explore other potential mechanism to develop a novel approach to delay the epileptic progression. Since approximately 30 years ago, histone deacetylases (HDACs), the versatile epigenetic regulators responsible for gene transcription via binding histones or non-histone substrates, have grabbed considerable attention in drug discovery. There are also substantial evidences supporting that aberrant expressions and/activities of HDAC isoforms are reported in epilepsy and HDAC inhibitors (HDACi) have been successfully utilized for therapeutic purposes in this condition. However, the specific mechanisms underlying the role of HDACs in epileptic progression have not been fully understood. Herein, we reviewed the basic information of HDACs, summarized the recent findings associated with the roles of diverse HDAC subunits in epilepsy and discussed the potential regulatory mechanisms by which HDACs affected the development of epilepsy. Additionally, we also provided a brief discussion on the potential of HDACs as promising therapeutic targets for epilepsy treatment, serving as a valuable reference for basic study and clinical translation in epilepsy field.

## Introduction

**Fig. 1:**
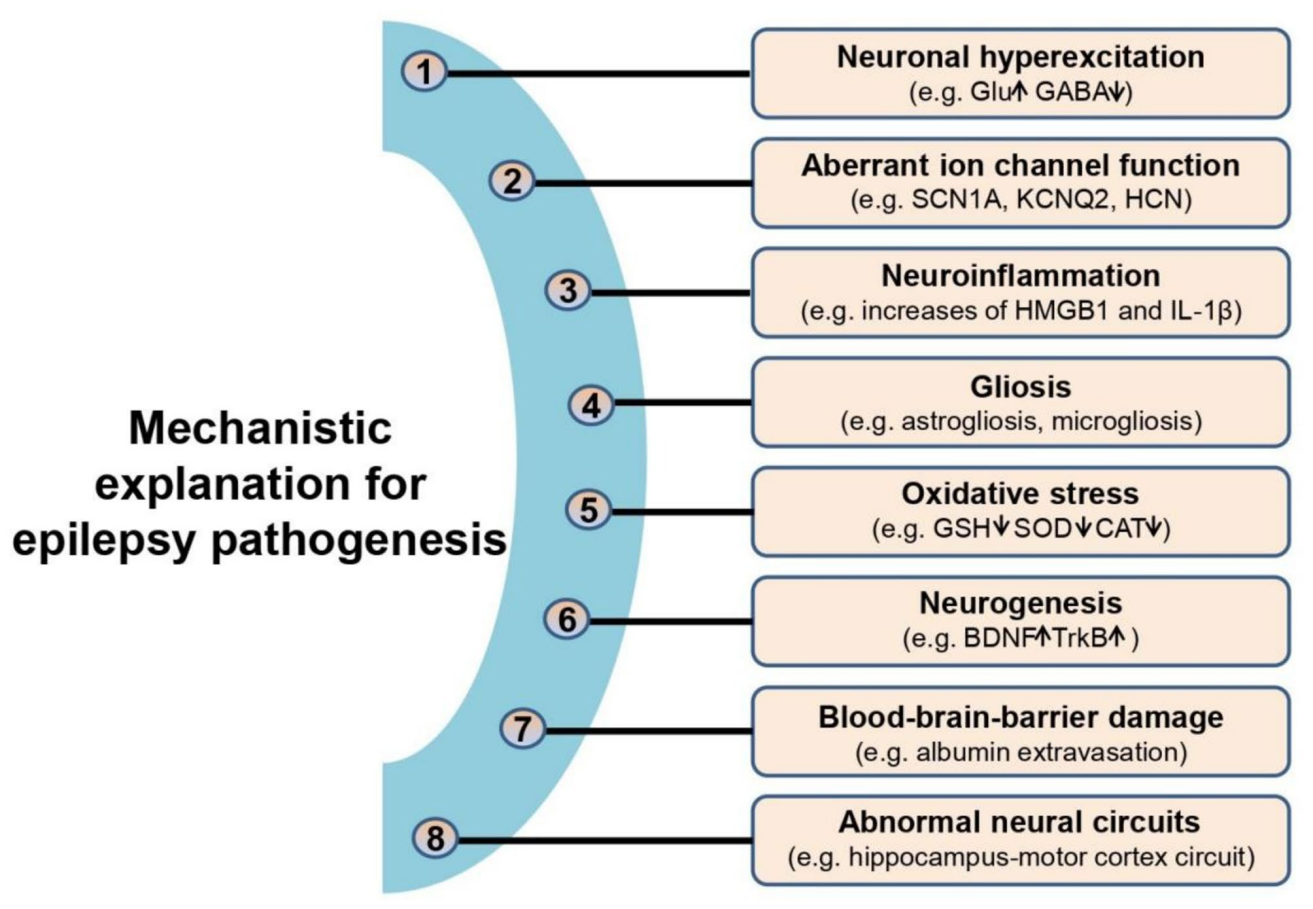
Mechanistic explanation for epilepsy. There are at least 8 types of mechanistic bases for explaining epilepsy pathogenesis, which includes neuronal hyperexcitation (e.g. elevation of glutamate, decrease of GABA), aberrant ion channel function (e.g. SCN1A, KCNQ2, HCN), Neuroinflammation (e.g. increases of HMGB1 and IL-1β), gliosis (e.g. astrogliosis, microgliosis), oxidative stress (e.g. decreases of GSH, SOD and CAT), Neurogenesis (e.g. increases of BDNF and TrkB) and Blood-brain-barrier damage (e.g. albumin extravasation) and abnormal neural circuits (e.g. hippocampus-motor cortex circuit). These causes may sometimes simultaneously involve in the development of epilepsy. *Note*: GABA, γ-aminobutyric acid; HMGB1, high mobility group box 1; GSH, glutathione; SOD, superoxide dismutase; CAT, catalase; BDNF, brain-derived neurotrophic factor

Epilepsy is among the most widespread chronic debilitating neurological disorders, afflicting approximately 70 million people globally, i.e., 1–2% of world’s population [1, 2]. Multiple contributing factors have been identified for epilepsy, which include structural abnormalities, genetic mutations, acute brain injuries (e.g., neurotrauma, stroke or brain tumors), autoimmune conditions, central nervous system (CNS) infections, and metabolic disturbances [3, 4]. Nearly half of the patients, however, have unrecognized etiopathogenetic origins [5]. Nowadays, there are a variety of mechanistic explanations for epilepsy as summarized in Fig. 1, which includes neuronal hyperexcitation [6], aberrant ion channel function [7], neuroinflammation [8, 9], gliosis [10], oxidative stress [11], neurogenesis [12], blood-brain-barrier damage [13] and abnormal neural circuit [14]. Regardless of various disease-inducing mechanisms, epilepsy is characterized by aberrant, recurrent and hypersynchronous discharge of a large population of cerebral neurons, usually accompanied with aberrant electroencephalogram (EEG), behavioral deficits and histopathologies [10, 15]. Seizures are prevalent behavioral changes that encompass a variety of clinical manifestations, such as convulsions, loss of consciousness, myoclonus and muscular hypotonia [5]. On the basis of an official definition promulgated by the 2017 International League Against Epilepsy (ILAE), epilepsy can be divided into four categories, namely generalized epilepsy, focal epilepsy, combined generalized and focal epilepsy, and unknown epilepsy [5]. In addition, it is also accompanied with diverse neuropsychiatric comorbidities, such as cognitive deficits, anxiety, depression and autism spectrum disorders [16, 17], which generates a serious health threat and creates an enormous economic burden on individuals and society. With respect to epilepsy treatment, drug therapy, surgery and ketogenic diets are classical methods for counteracting seizures [1]. The ketogenic diets, which are food groups containing high-fat, low-carbohydrate, and moderate-protein diets, have also been successfully utilized for the treatment of drug-resistant epilepsy (DRE) for decades [18]. However, recent investigations have illustrated that intake of ketogenic diets may result in various side effects such as cognitive decline and risk of cardiovascular events [19, 20], despite unknown mechanism. Surgical resection of epileptogenic zone can attain long-term seizure freedom in intractable epilepsy. However, it is often confronted with risks and postoperative complications [21]. To date, pharmacotherapy is the mainstay of clinical treatment for controlling seizures [22]. It is estimated that more than 30 anti-epileptic drugs (AEDs) have been licensed for curing epilepsy. Nevertheless, there are still around 30% of patients who respond poorly to the current AEDs [23]. Besides, the conventional AEDs only provide epilepsy symptom relief rather than exerting disease-modifying effect [24]. Moreover, long-term exposure of these AEDs often triggers adverse effects including hepatic injury and renal toxicity, cognitive deficit and psychiatric abnormalities [25]. Thus, elucidating the etiology of epilepsy is indispensable for the development of promising strategies that can modify the disease progression to improve the quality of life.

**Fig. 2:**
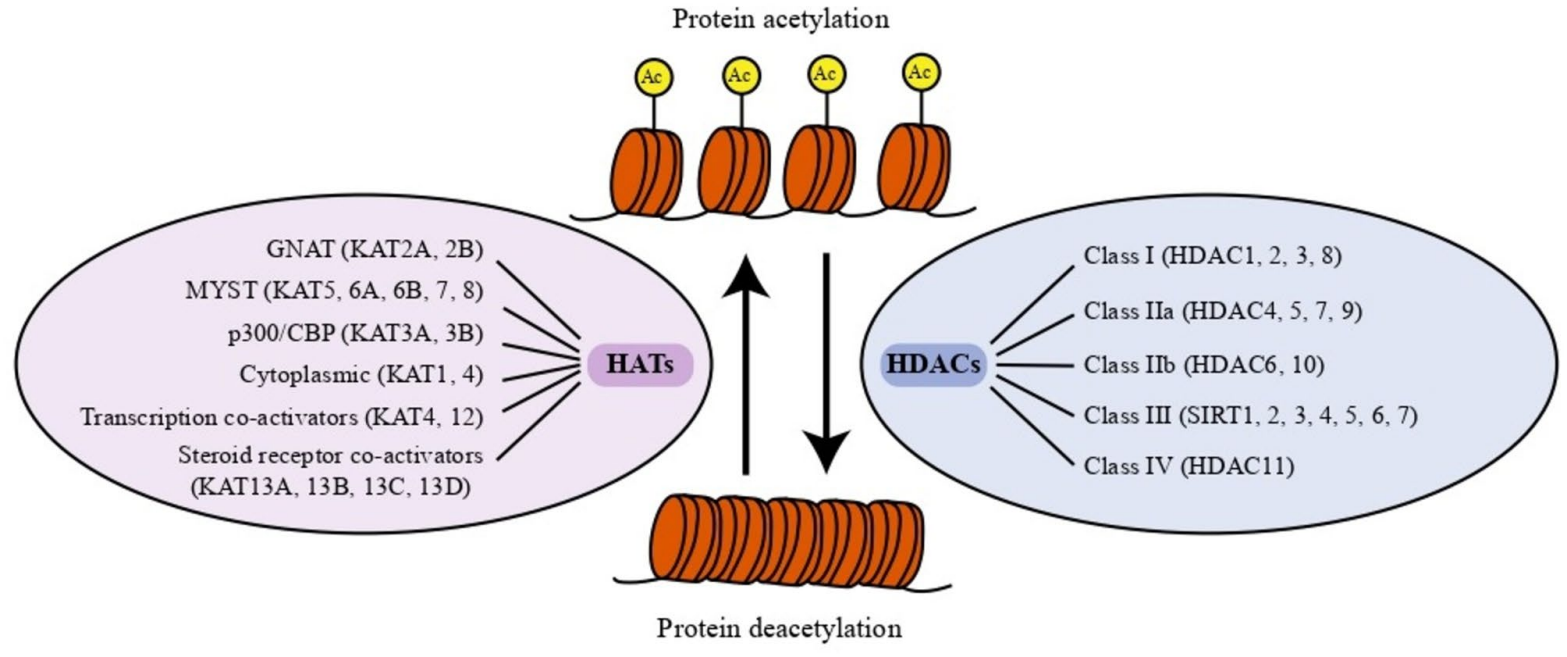
Overview of protein acetylation by histone acetyl-transferases (HATs) and protein deacetylation by histone deacetylases (HDACs). HATs and HDACs are epigenetic enzymes responsible for protein acetylation and protein deacetylation, respectively. Generally, HATs include six categories such as GNAT (KAT2A, 2B), MYST (KAT5, 6 A, 6B, 7, 8), p300/CBP (KAT3A, 3B), cytoplasmic (KAT1, 4), transcription co-activators (KAT4, 12) and steroid receptor co-activators (KAT13A, 13B, 13 C, 13D) while HDACs are composed of four classes of isozymes such as class I (HDACs 1, 2, 3 and 8), class IIa (HDACs 4, 5, 7 and 9), class IIb (HDACs 6 and 10), class III (also called sirtuins (SIRTs), including SIRT1-7) and class IV (HDAC11)

Repetitive seizures always lead to alterations in gene expressions, thus causing aberrant neural circuits, and ultimately affecting epileptic phenotype. Some of altered gene expressions are manipulated by epigenetic modifications [26, 27]. Epigenetics refers to stable alterations in gene expression without influencing DNA sequence [28], usually including DNA methylation [29], histone modification and RNA modifications [30–32]. Since epigenetic alterations are involved in a variety of human diseases including epilepsy [33, 34], epigenetic targets have attracted considerable attention in the field of drug discovery in recent years. In particular, histone deacetylases (HDACs), critical regulators responsible for post-translational modifications, are rapidly emerging (Fig. 2) [35]. By eliminating acetyl groups from the lysine residues of proteins, HDACs not only inhibit gene transcription, but also cause alternative post-translational lysine modifications including methylation, ubiquitination, and sumoylation [36]. Available reports have demonstrated that the increase of HDACs activity triggers protein deacetylation, leading to the pathogenesis of a plethora of human diseases including epilepsy [37, 38] and memory deficits [39]. Accordingly, pharmacological intervention of HDACs has a promising therapeutic potential against epilepsy. In recent years, various kinds of inhibitors of HDACs (HDACi) have proved to be beneficial for cancer treatment, parasitic, inflammatory and other diseases [40, 41]. Nowadays, there are four HDACi approved for curing cancer, which include Vorinostat (SAHA) [42], Romidepsin (FK228) [43], Belinostat (PXD-101) [44], Chidamide [45] (for cutaneous T-cell lymphoma (CTCL) or peripheral T cell lymphoma (PTCL)) and Panbinostat (LBH-589) [46] (for multiple myeloma). Despite successful utilization of these HDACi in cancer, there are also investigations supporting the therapeutic potential of HDACi against epilepsy. The direct evidence comes from the study showing that valproic acid (VPA) acts as a classical HDACi and achieves great benefits on diverse seizure types [47]. Additionally, lacosamide, an approved drug for treating epilepsy, has also been reported to induce histone hyperacetylation via reductions of HDACs protein expressions, enhancement of histone acetyltransferase or HDAC inhibition by its metabolites [48, 49]. Furthermore, clinical trials on the evaluation of some other HDACi including divalproex sodium (DVPX) and suberoylanilide hydroxamic acid (SAHA, also named Vorinostat) are also conducted to test the therapeutic potential against patients subject to epilepsy. These pioneering studies suggest targeting HDACs shows promise in the treatment of epilepsy.

In this review article, we aimed to update the understanding of the possible roles of HDACs in the pathophysiology of epilepsy, discuss the likely regulatory mechanisms of these enzymes and summarize the recent progress in the development of HDACi in epilepsy rodent models and patients. Additionally, we also gave future prospects on HDACs as epigenetic targets for epilepsy.

### An overview of HDACs

#### Classification of HDACs

HDACs (also called lysine deacetylases, KDACs) superfamily, evolutionarily conserved enzymes occurring in yeast cells and higher eukaryotic organisms [50], contains 18 known isoforms and can be divided into four classes [51]. Among these proteases, class I (HDAC1/2/3/8), class IIa (HDAC4/5/7/9), class IIb (HDACs 6/10) and class IV (HDAC11) are zinc-dependent family of enzymes, while class III (also called sirtuins (SIRTs), including SIRT1-7) are nicotinamide adenine dinucleotide (NAD^+^)-dependent deacetylases [52], as summarized in Table 1. The numbering of HDAC isoform is based on the chronological order of discovery. For instance, HDAC1 was firstly discovered several months before HDAC2, despite both in 1996 [53, 54] and in the next year HDAC3 was identified [55]. HDACs have distinct distribution patterns in diverse brain regions. There are widespread distributions for HDACs in a variety of cells notably neurons and subcellular analysis reveals that the proportion in the nucleus is more prominent than that in the cytoplasm [56]. Concerning class I HDACs, except for the distributions of HDAC3 both in the nucleus and cytoplasm, other enzymes in this category exhibit a rich expression in the nucleus. Class IIa HDACs can shuttle between the nucleus and cytoplasm following diverse cell signal stress while the distributions of class IIb HDACs are mainly detected in the cytoplasm [57]. Class III HDACs, which plays an essential role in metabolic regulation, have a wide distribution in the nucleus, cytoplasm, and mitochondria [58]. The class IV HDAC including a single HDAC, namely, HDAC11, localizes to the nuclear compartment and functions as a regulator in immune cell fate although the concrete mechanism remains uncharacterized [51].

**Table 1:** The classification of HDACs and their binding substrates of HDACs

			Substrates	
Category	Zn^2+^/NAD^+^	HDAC isotype	Histone substrates	Non-histone substrates
Class I	Zn^2+^-dependent	HDAC1	H3 histone (K9ac, K14ac, K18ac, K23ac, K27ac)	p53 (K382ac), HSP70, STAT3, E2F-1
		HDAC2	H3 histone (K9ac), H4 histone (K12ac)	p5 (K320ac), Nrf2, Bcl-6, STAT3
		HDAC3	H3 histone (K9ac, K14ac), H4 histone (K5ac, K12ac)	p65 (K310ac, K314ac, K315ac), MEF2, STAT1, STAT3,GATA1, NF-κB
		HDAC8	H3 histone (K9ac, K14ac, K56ac)	ARIDIA (K1808ac), cortactin
Class IIa	Zn^2+^-dependent	HDAC4		p53, STAT1, SRF, HIF-1α, ATF4, Foxo1, Runx2
		HDAC5		reelin
		HDAC7		HIF-1α
		HDAC9		
Class IIb	Zn^2+^-dependent	HDAC6	H3 histone (K14ac), H4 histone (K5ac, K12ac, K16ac)	G3BP1 (K376ac), cortactin (K124ac), HSP90 (K294ac), β-Catenin, PrxI, PrxII, Survivin, AKT (K37ac, K163ac)
		HDAC10		MSH2, MMP2, MMP9, HSP70
Class III	NAD^+^-dependent	SIRT1	H3 histone (K9ac, K14ac, K56ac), H4 histone (K16ac)	TGF-β, PGC-1α, p53 (K379ac)
		SIRT2	H3 histone (K56ac), H4 histone (K16ac)	G6PD, α-tubulin
		SIRT3	H3 histone (K4bhb, K4cr, K9bhb, K18bhb, K23bhb, K27bhb), H4 histone (K16ac, K16bhb)	ACSS1 (K642ac), SDHA (K179ac), Ku70, Nampt, MRPL10
		SIRT4		MCCC, MCD, GDH
		SIRT5		PKM2 (K311ac), CPS1
		SIRT6	H3 histone (K9ac, K56ac)	TNF (K19ac, K20ac)
		SIRT7	H3 histone (K18ac)	FKBPS1 (K28ac, K155ac), Ran (K37ac), U3-55 K (K12ac, K25ac)
Class IV	Zn^2+^-dependent	HDAC11		IL-10

*Note* NAD^+^, nicotinamide adenine dinucleotide; HSP70, heat shock protein 70; STAT3, signal transduction and activation of transcription 3; SIRT 1–7, sirtuin 1–7; Nrf2, NF-E2-related factor 2; MEF2, myocyte enhancer factor-2; SRF, serum response factor; HIF-1α, hypoxia-inducible factor 1α; ATF4, activating transcription factor 4; PrxI, peroxiredoxin I; MMP2, matrix metalloproteinase 2; TGF-β, transforming growth factor-β; PGC-1α, peroxisome proliferators-activated receptor-1α; G6PD, glucose-6-phosphate dehydrogenase; ACSS1, acetyl-CoA synthetase short-chain subfamily member 1; SDHA, succinate dehydrogenase subunit A; Nampt, nicotinamide phosphoribosyltransferase; MRPL10, mitochondrial ribosomal protein L10; PKM2, pyruvate kinase 2; TNF, tumor necrosis factor

#### Substrates of HDACs

The traditional concept supports that HDACs mainly repress gene transcription by deacetylation of histones through removing acetyl groups from their lysine residues. However, recent work has shown that, in addition to using histones as substrates for deacetylation, HDACs can also inhibit the acetylation of non-histone proteins and modulate diverse neurobiological processes [59, 60]. Below, we outline histone and non-histone substrates binding by HDACs.

*Histone Substrates.* Histones including H1, H2A, H2B, H3 and H4 are known positively charged proteins which can bind tightly to the negatively charged DNA, favoring histone-DNA complex formation (also named nucleosome) [61]. It is well established that HDACs-mediated histone deacetylation contributes to condensed chromatin state, allowing suppression of gene-specific transcription. Class I HDACs and class IIb HDACs are considered as representative transcription repressors. For instance, HDAC1, HDAC2, and HDAC3, which belong to class I HDACs, have been demonstrated to mediate the deacetylation of mitogen-activated protein (MAP) kinase phosphatase 1 (MKP1), resulting in elevation of MAP kinase (MAPK) and suppression of innate immune signaling [62]. In the aspect of class IIb HDACs, Fischle and colleagues reveal that neither HDAC4 nor HDAC5 exhibits deacetylation activity. However, when combination with HDAC4 (or HDAC5) with HDAC3, the transcriptional repression is facilitated via simultaneous binding of silencing mediator for retinoid and thyroid receptors (SMRT) and nuclear receptor corepressor (N-CoR) [63, 64]. These data implicate that transcription suppressive effects of HDACs can target distinct genes. HDACs-mediated histone deacetylation is involved in the regulation of a plethora of biological processes (e.g. metabolism, inflammation and immunity, and cell survival) [65]. In detail, inhibition of HDAC by sodium butyrate induces acetylation of histone H3 (at Lys14) and histone H4 (at Lys16), resulting in the activation of the transcriptional regulation of CYP1A1, an important metabolic enzyme, in human colon carcinoma HCT116 cells [66]. In macrophages, it has been demonstrated that HDAC4 knockdown decreases histone H3K9/K14 acetylation and attenuates lipopolysaccharide (LPS)-induced inflammatory gene expression, indicating the pro-inflammatory role of HDAC4 [67]. Additionally, deficiencies of HDAC1 and HDAC2 can reduce cell survival via decreasing the acetylation status of H3K56ac, H4K16ac and H4K91 [68], suggesting the positive effects of HDAC1 and HDAC2 on cell survival.

*Non-Histone Substrates.* Beyond deacetylation of histone proteins, HDACs also accept non-histone proteins as their substrates, finally affecting physiological and disease-related cellular processes, such as transcriptional machinery, DNA damage, signal transduction, protein folding, autophagy, and metabolism [60]. At least two categories of non-histone substrates are deacetylated by HDACs, including transcription factors and non-transcription factors. The representative transcription factors mediated by HDACs include p53, signal transduction and activation of transcription 1 (STAT1), STAT3, GATA-binding factors (e.g. GATA1/2) and nuclear factor κB (NF-κB) [69] while non-transcription factors for HDACs-mediated deacetylation contain cytoskeletal protein (e.g. α-tubulin), molecular chaperone (e.g. the molecular chaperone heat shock protein 90 (Hsp90) and cohesion (e.g. SMC3) [70]. Generally, HDACs-mediated deacetylation has physical and pathophysiological roles. There are various evidences supporting that yperacetylation of p53 is suppressed by HDAC1 and HDAC2 [71] in embryonic epidermis. In hematopoietic stem cells, it has also been demonstrated that GATA-2 was transcriptionally silenced via HDAC3-meidated deacetylation [70]. These data suggest that deacetylation by HDACs plays a critical role for maintenance of physical function. In the meantime, there are also substantial evidences supporting the importance of HDACs-mediated deacetylation in disease conditions. For instance, HDAC7 was found to trigger the deacetylation of STAT3 and suppress the gene activation, finally promoting lung tumorigenesis [72]. In the breast cancer cell line MCF-7, it has also been shown that SMC3 acetylation of cohesion is activated after HDAC8 inhibition [73]. The impressive data supporting the role of deacetylation by HDACs in epilepsy arises from the study that the interaction of HDAC3 with MADS-box domain of MEF2 results in MEF2 deacetylation in nucleus and the decrease of MEF2 transcription [74].

Collectively, our summarizations shown above depict that HDACs can regulate the deacetylations of both histone proteins and non-histone substrates.

### Beyond deacetylation: the non-deacetylase functions of HDACs

In addition to the protein deacetylation for HDACs, there are also several evidences showing that HDACs possess deacetylase-independent functions such as sumoylation, decrotonylation, ubiquitin-dependent function and lactylation eraser as shown below.

*Sumoylation*. Posttranslational modification with small ubiquitin-like modifier (SUMO) is a key regulatory protein modification in mammals, which is characterized with covalent conjugation of SUMO to specific lysine residues of target proteins [75, 76] and regulates diverse cellular processes [77]. Prior study showed that deficiency of Sentrin/SUMO-specific protease 2 (SENP2) resulted in hyper-SUMOylation of multiple potassium channels and neuronal hyperexcitability, finally triggering spontaneous seizures and sudden death [78, 79], which suggests that sumoylation is vital for epilepsy. HDAC4 and HDAC7 have been intensively demonstrated to possess SUMO E3 ligase activity. For instance, HDAC4 can stimulate the sumoylation of several targets including MEF2 and zinc finger X-linked duplicated (ZXD) family zinc finger C (ZXDC). In particular, the contribution of HDAC4 to the sumoylation for MEF2 finally leads to transcription inhibition [80] while HDAC4-mediated sumoylation of ZXDC activates the transcription activity [81]. Meanwhile, HDAC7 cooperated with the E2 SUMO ligase, Ubc9, was also found to facilitate promyelocytic leukemia protein (PML) sumoylation and the formation of PML nuclear bodies [82], suggesting the SUMO-like function of HDAC7. Although no direct evidence has been found with the aspect of HDACs-mediated sumoylation in epilepsy, there are multiple results indicating the role of sumoylation by HDACs in other neurological conditions. For instance, HDAC4 is observed to bind SUMO-conjugating enzyme Ubc9, regulating the long-term memory formation of fruit flies [83]. Additionally, HDAC1 SUMOylation is also found to protect against Aβ toxicity and alleviate AD [84]. Taken together, it is worthy to explore the role of HDACs-mediated sumoylation in epilepsy.

Decrotonylation. Besides deacetylation, there are also other types of PTMs identified in histone lysine (K) residues, which include crotonylation, malonylation and butyrylation [85, 86]. Recently, histone crotonylation has grasped profound attention due to the broad distribution in all core histones, which serves as a promoter or potential enhancer [85]. Four subunits of class I HDACs (i.e. HDAC1/2/3/8) exhibit a potent decrotonylation activity [87]. Further work reveals that class I HDACs-catalyzed decrotonylation represents the major histone decrotonylases in mammalian cells [85]. Recent investigation has also illustrated pharmacological inhibition of HDAC by butyrate, a critical short chain fatty acid (SCFA) derived from the gut microbiota, promotes histone crotonylation in the colon and depletion of the gut microbiota reduces the generation of SCFA, resulting in a loss of histone crotonylation [88]. It implicates that microbiota-derived SCFA facilitates histone crotonylation in the colon at least in part via HDACs. Until now, there is no report on HDACs-mediated decrotonylation in epilepsy. However, in hypoxic-ischemic encephalopathy, a common neurological condition in pediatrics, it has been demonstrated that treatment with HDACi sodium butyrate facilitates H3K9 crotonylation and causes the activation of neurotrophic genes [89]. These data implicates that HDACs-associated decrotonylation is vital for the regulation of neural function.

*Ubiquitin-dependent function*. HDAC6 is a well-recognized protease for exerting ubiquitin-related function. In detail, elevation of HDAC6 promotes the formation of aggresome, which emerges as a cytoprotective response in the clearance of toxic misfolded protein aggregates found in neurodegenerative disease such as Parkinson’s disease [90], via binding polyubiquitinated misfolded protein including CFTR-ΔF508 [91]. It suggests that HDAC6 displays a ubiquitin binding capacity and facilitates aggresome formation, representing a protective strategy against neurological disorders. In addition, the promotion of HDAC1 to SMAD7 ubiquitination [92, 93], thereby decreasing the half-life of the protein, is also observed, although the concrete mechanism remains unknown. There is rare data reporting HDACs-mediated ubiquitination modification in epilepsy. However, since the vital role of this phenomenon in other neurological dysfunction such as Parkinson’s disease as mentioned above, it is essential to explore the concrete role of ubiquitination by HDACs for epileptic progression.

*Lactylation eraser.* Lactylation is a newly discovered type of epigenetic modification found in lysine residue of histone protein by the research group of Zhao in 2019 [94], which can directly regulate gene transcription. Via screening 18 HDACs for the potential delactylase activity, Zhao and colleagues reported that HDAC1-3 and SIRT1-3 could cleave L-lactyllysine marks in vitro [95], especially for nuclear HDAC1-3 serving as the most robust delactylases, suggesting a critical role of HDAC1-3 for the removal of histone lactylation [95]. As it was previously demonstrated that lysine lactylation was induced by neural excitation, a key molecular mechanism for the development of epilepsy [96], with the detailed results showing the positive association of increase of lysine lactylation and the expression of the immediate-early gene marker of neuronal activity c-Fos [97], it is hypothesized that histone delactylation by HDAC1-3 may inhibit the development of epilepsy and prevent seizures. As a result, antiepileptic medication can be proposed to target HDAC1-3-mediated histone delactylation. Further research is still required to explore the detailed role of HDAC1-3 in histone lactylation and neuronal excitotoxicity during epilepsy.

### Role of HDACs in epilepsy

**Fig. 3:**
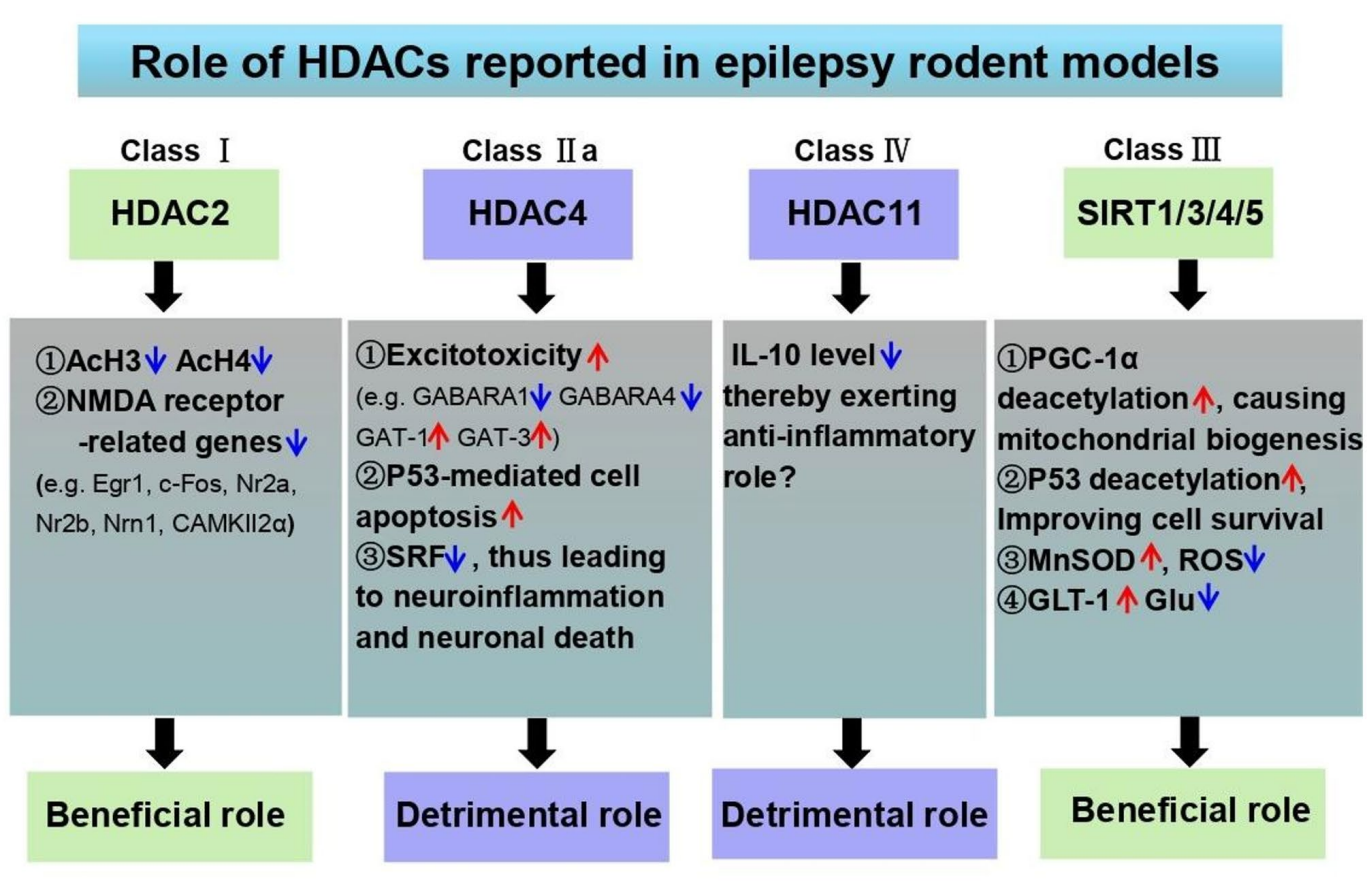
HDACs mediate pathological mechanism of epilepsy. There are at least four types of HDACs reported to be vital for epilepsy, which include class I (i.e. HDAC2), class IIa (i.e. HDAC4), class IV (i.e. HDAC11) and class III HDACs (i.e. SIRT1, 3, 4, 5). In detail, activation of HDAC2 results in the acetylation of histone 3 and histone 4 and down-regulations of NMDA receptor-related genes including Egr1, c-Fos, Nr2a, Nr2b, Nrn1, CAMKII2α, indicating the protective role in epilepsy. In aspect of HDAC4 belong to class IIa HDAC family, it has versatile roles for the exacerbation of epileptic progression via promotion of neuronal excitotoxicity through decreasing GABAergic signaling, p53-mediated neuronal apoptosis and neuroinflammation through blocking the transcription of SRF. The member of class IV HDAC family HDAC11 possibly has the anti-inflammatory role in epilepsy via decreasing IL-10 expression although the concrete mechanism remains unknown. In terms of class III HDAC family especially SIRT1, 3, 4 and 5 reported in epilepsy research, it has demonstrated that distinct roles of each isotype are observed (SIRT1 for PGC-1α deacetylation-mediated mitochondrial biogenesis and p53 deacetylation-mediated cell survival, SIRT3 for inhibition of ROS via activating MnSOD, SIRT4 for decrease of glutamate via increasing GLT-1 and SIRT5 for alleviation of hippocampal neurodegeneration), however, the overall beneficial effects for epileptic relief. *Note*: NMDA, N- methyl-d-aspartate; GABA, γ-aminobutyric acid; GABARA1, A type GABA receptor α1; GABARA4, A type GABA receptor α4; GAT-1, GABA transporter 1; GAT-3, GABA transporter 3; SRF, serum response factor; PGC-1α, peroxisome proliferator-activated receptor γ coactivators 1α; ROS, reactive oxygen species; GLT-1, glutamate transporter-1

Although the pathogenesis of epilepsy has not been fully established, excitotoxicity, oxidative stress and neuroinflammation are regarded as the triads of epileptogenesis, a process during which triggers of brain insults such as status epilepticus by chemoconvulsants, infection or cerebrovascular disease include and the brain undergoes a cascade of morphological and functional changes prior to the onset of spontaneous recurrent seizures in chronic epilepsy [98], and epilepsy-associated neurodegeneration. In the meantime, interrupted transcriptional regulation often occurs in the susceptibility and progression of various CNS diseases, including epilepsy [99]. As such, HDACs, a group of vital epigenetic targets manipulating gene transcription, are probably involved in the occurrence of epilepsy. As outlined below, a number of class I, class II and class IV HDACs are implicated in the setting of epilepsy (Fig. 3).

*Class I HDACs.* Several studies have demonstrated the obviously altered expressions of class I HDACs including HDAC1, HDAC2, HDAC3 and HDAC8 in mouse TLE models [37, 100]. In details, the transcriptional levels of HDAC1 and HDAC2 are decreased in the granule cells and the pyramidal cell layer of hippocampus in the acute stage of KA-induced epilepsy. These two isoforms exhibit also similar tendency in Pilo-induced acute epileptic seizure while HDAC3 and HDAC8 are significantly reduced at the mRNA level in the chronic epilepsy [37]. In the two epilepsy models as mentioned above, the decrease of class I HDACs initiates the acetylation of histone H4 protein in rat hippocampal neurons and thereby activates the factors involved in epileptogenesis such as immediate early genes (IEGs) (e.g. c-fos and c-jun) and growth factors (e.g. brain-derived neurotrophic factor (BDNF)) [101]. These data suggest that class I HDACs display a neuroprotective effect. This notion is also further supported by the report illustrating that activation of HDAC1 promotes neuroprotection against human neurons during neurodegeneration [102]. In the aspect of HDAC2, there is also consistency showing that repeated electroconvulsive seizures treatment causes the up-regulation of HDAC2 expression both at the mRNA and protein levels in the rat frontal cortex, which further reduces the histone acetylation of H3 and H4 proteins and diminishes NMDA receptor-related genes involved in the development of epilepsy [103]. Treatment with a pan-HDAC inhibitor sodium butyrate reversed this phenomenon. Thus, these results support the hypothesis that HDAC2 activation has a protective role for seizure susceptibility.

*Class II HDACs.* HDAC4, an important member of class II HDACs, has been extensively discussed in epilepsy. HDAC4 is abundantly expressed in brain tissues, predominately in the cytoplasm of neurons through binding to the 14-3-3 protein [104]. Both of the interaction involves HDAC4 phosphorylation. It has been demonstrated that the promotion of HDAC4 phosphorylation is found after overexpression of CDKL5 via facilitating the cytoplasmic retention of HDAC4 while loss of CDKL5 leads to the decrease of HDAC4 phosphorylation, ultimately increasing the translocation of HDAC4 into the nucleus [105]. These data indicate that CDKL5 plays a key role for HDAC4 to travel between the cytoplasm and the nucleus. MEF2 is also reported to nucleocytoplasmic switch via interaction with HDAC4 [104]. In the nucleus, protein phosphatase 2 A (PP2A)-mediated dephosphorylation at serine 298 residue affects the interaction of the nuclear localization signal of HDAC4 and MEF2, finally altering the neuronal activity. Besides, neuronal activity is also of vital importance for the nucleocytoplasmic shuttling of HDAC4 since previous work reveals that spontaneous neuronal activity contributes to nuclear export of HDAC4 [104]. In vitro, HDAC4 exacerbates excitotoxic insults in retinal ganglion cells (RGCs) and inhibition of HDAC4/5 by LMK-235 prevented RGCs degeneration [106]. It indicates that activation of HDAC4 promotes neuronal excitotoxicity, a hallmark of epilepsy. Further research is indispensable for exploration the potential mechanism of HDAC4 following epileptogenesis. In contrast, neuronal inhibition mediated by gamma-aminobutyric acid (GABA) signaling pathway is also affected by HDAC4. It has been discovered that knockdown of HDAC4 attenuates epileptic seizures in rats via up-regulations of GABA_A_ receptors including GABA_A_Rα1 and GABA_A_Rα4 and down-regulations of GAT-1, and GAT-3, finally activating GABA-mediated synaptic inhibition [107]. These results implicate that targeting HDAC4 may become a promising approach for the treatment of epilepsy. Oxidative stress is also important for HDAC4 as there are substantial evidences supporting that elevation of ROS production results in the increase of nuclear localization of HDAC4. In the nucleus, HDAC4 always binds to various targets such as p53 and serum response factor (SRF). It has been illustrated that p53 serves as a critical non-histone protein substrate of HDAC4 and involves in neuronal death signaling following epileptic seizures [108]. The up-regulation of p53 is positively associated with neuronal apoptosis in the hippocampus of patients with epilepsy [109] and the neuronal death can be evidently blocked by p53 antagonization [110]. However, there is discrepancy showing that targeting p53 may cause a detrimental response following epileptogenesis [111]. Collectively, it indicates that HDAC4-p53 interaction functions as an intriguing target due to the inhibition of p53 caused by HDAC4-mediated deacetylation. Another widespread nuclear target of HDAC4 is the SRF which is a key regulator for mediating IEGs such as c-Fos and Egr1 [112]. Recent studies have indicated that HDAC4 profoundly suppresses SRF-mediated transcription. Deletion of SRF has been discovered to contribute to several processes involving in epileptogenesis, such as neuroinflammation, mossy fiber sprouting, and cell death [113, 114]. Although there are studies supporting the nuclear localization of HDAC4-SRF complex in several cells, there is still a need to gain a better understanding in the case of epileptic/epileptogenic neuronal cells.

*Class IV HDACs.* HDAC11 is a single member of class IV HDACs. Overexpression of HDAC11 negatively regulates IL-10 expression [115]. In such a scenario, HDAC11 exhibits an immunomodulatory function. Elevation of HDAC11 is observed in patients with hippocampal sclerosis (HS) which are positively related to the down-regulation of IL-10 in mesial temporal lobe epilepsy with hippocampal sclerosis (MTLE-HS) patients [116]. These data give the hints that HDAC11/IL-10 complex may serve as a potential therapeutic target, although more convincing findings are required.

There are also some other isoforms of HDACs which are potentially important for epilepsy. For instance, the alterations of SIRTs in epilepsy are widely appreciated. Recent studies have revealed the elevation of SIRT1 in a rat model of status epilepticus, a common neurological emergency which is defined as a prolonged seizure with at least five minutes or multiple seizures with incomplete recovery of consciousness [117] and its neuroprotection is conducted via deacetylation of proliferator-activated receptors and γ coactivator 1α (PGC-1α), an important regulator for mitochondrial biogenesis [118, 119]. The contribution of SIRT1 to cell survival is also found as it has the capacity to deactivate p53, a critical factor for neuronal death as discussed above following epileptogenesis. In addition, some other members of SIRTs including SIRT3, 4 and 5 display neuroprotective roles against epilepsy as a result of evidences supporting the improvement of neuronal damage for SIRT3 via scavenging ROS in mitochondria [120], protection from excitotoxicity for SIRT4 via increasing GLT-1-mediated glutamate uptake [121] and reduction of hippocampal neurodegeneration and attenuation of seizure susceptibility for SIRT5 [122]. In mitochondria, SIRT4 is reported to not only suppress malonyl-CoA carboxylase to inhibit fatty acid oxidation, but also repress pyruvate dehydrogenase and stimulate mitochondrial ATP production [123]. SIRT5 functions as a global regulator of mitochondrial lysine succinylation and involved in metabolic regulation [124]. Taken together, these results indicate the crucial role of histone acetylation in epileptogenesis. However, all above data are obtained after seizure onset, which may represent the consequence of epileptic seizures rather than the cause. Further studies are warranted to detect the alterations before the occurrence of seizure and following the time course of the disease progression. The reasons why different subtypes of HDACs have distinct effect on epilepsy are possibly due to impact on different downstream target molecules and neural circuits across distinct brain regions. In addition, regarding the relationship of HDACs expression pattern and the circuits known to be susceptible to seizures, there is scarce data until now. However, a prior work demonstrated that SIRT1 modulated neural activity-related genes such as glutamatergic and GABAergic receptor gene expression [125], which are vital for neural circuit formation in epilepsy. Besides, HDAC6 was also reported to promote the growth of dendrite [126], a critical factor for neural circuit formation. These evidences indicate that HDACs plays a critical role in the regulation of neural circuits, which subsequently affects seizure generation and epileptic progression. The concrete role and regulatory mechanism of HDACs in circuit formation are essential to further explore in the future.

### Pharmacological effects of HDACi in preclinical studies

**Fig. 4:**
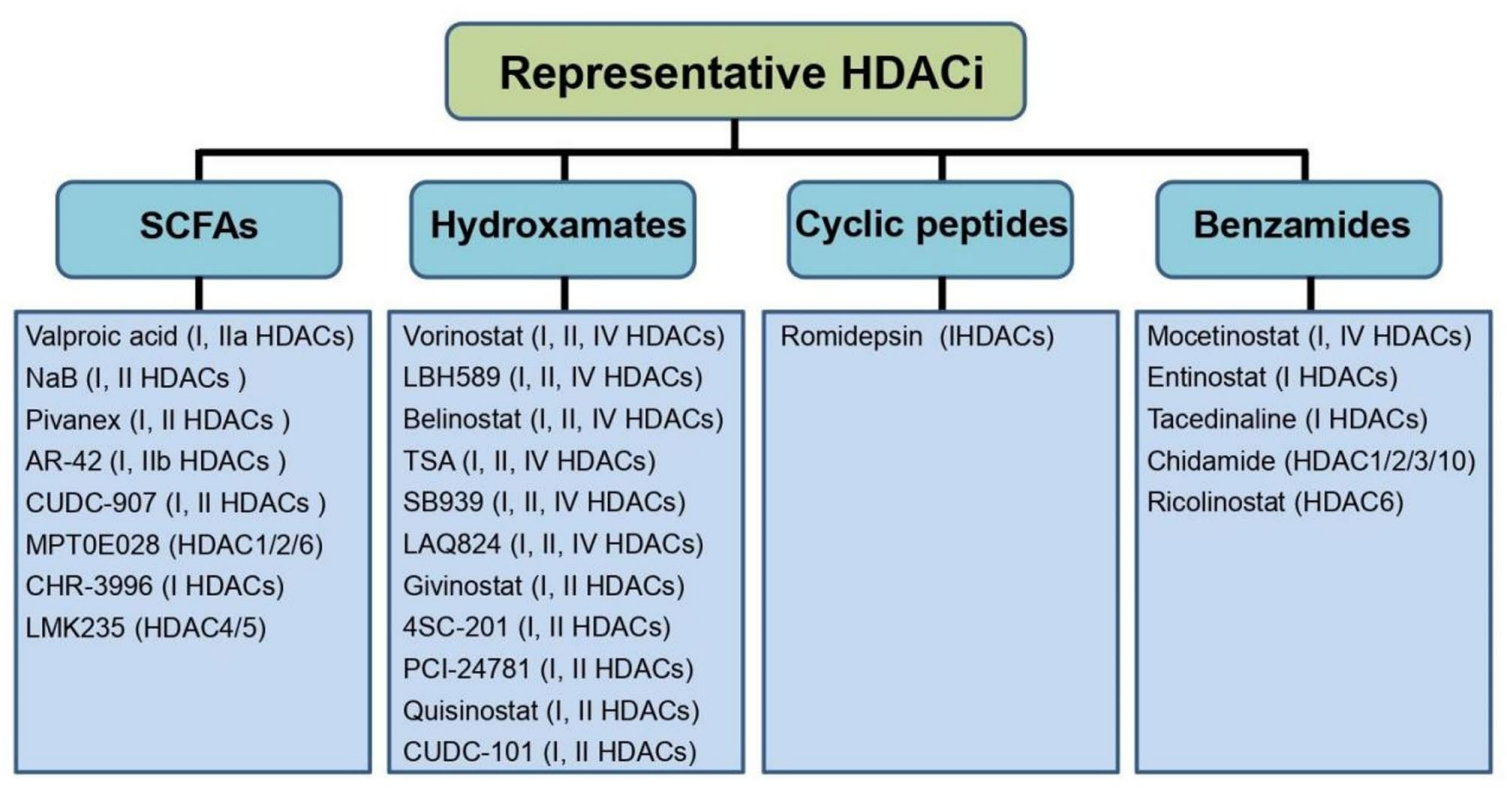
Classification of HDACi and representative HDACi. There are four categories of HDACi, namely, SCFAs, hydroxamates, cyclic peptides and benzamides according to their unique chemical structures. The representative HDACi in each sort are displayed. *Note*: SCFAs, short chain fatty acids

HDACi are powerful tools for the pharmacological intervention of HDACs and may show promise in the treatment of various human diseases including epilepsy in clinics. Nowadays, large amounts of HDACi are obtained via artificial synthesis or extraction from natural resources. These agents always have variations in the aspect of target specificity and pharmacokinetic properties in preclinical studies and clinical settings. To the best of our knowledge, there are hitherto at least four categories of HDACi, namely, SCFAs, hydroxamates, cyclic peptides and benzamides according to their distinctive chemical structures [127], as summarized in Fig. 4. Each of these HDACi is likely to act on multiple HDACs, which is difficult to determine whether the effects of HDACi (also including toxicity) are achieved via inhibition of a specific HDAC isotype or combined abrogation of multiple HDACs [51].

Development of compounds that can inhibit HDACs began almost 30 years ago. Currently, they are emerged as promising anticancer drugs since substantial evidences support that they exhibit versatile effects such as inductions of apoptosis, differentiation and cell cycle arrest and inhibitions of migration, invasion, and angiogenesis in various cancer cell lines. In animal models and patients, these agents showed pronounced anti-tumor effect [128]. In addition, the effects of HDACi on epilepsy have also been explored in vitro and in rodent models (Table 2). Actually, there is a long history regarding the clinical application of HDACi for epilepsy [129, 130]. The convincing evidence supports that VPA, a branched SCFA deriving from valeric acid, serves as an inhibitor targeting multiple HDACs especially class I and class IIa HDACs and has been used for more than 50 years to provide the satisfactory relief of epileptic seizures and other epileptic medications, particularly psychiatric comorbidities [131, 132]. The long-term epigenetic effects of VPA are reported to modulate gene transcription and synaptic receptors and several signaling molecules, which are involved in excitotoxicity and neuroprotection [133]. It has been demonstrated that VPA could down-regulate the expression of SCN3A, a gene coding the α-isoform of the voltage-gated sodium channel III, which were overexpressed in seizure animal models [134]. Epigenetic analysis revealed that VPA mainly induced the methylation at the − 39 C site in Scn3a promoter. Since up-regulation of SCN3A has been demonstrated to enhance neuronal excitability and contribute to the development of epilepsy, the reduction of SCN3A expression by VPA due to its promotion on methylation may underlie the anti-epileptic effect of VPA. It remains unclear whether VPA diminishes the expression of SCN3A via HDAC inhibition. However, there are some indirect evidences supporting that inhibition of HDAC by VPA can enable methylated DNA to be more accessible to DNA demethylase, leading to DNA demethylation [135], which suggests that the induction of the methylation of Scn3a promoter by VPA is likely to be affected by the regulation of HDAC. Furthermore, this phenomenon is exclusive for VPA as treatment with other anticonvulsants, such as carbamazepine or lamotrigine did not trigger alteration in the expression of SCN3A. Additionally, the inhibitory effect of seizure-induced neurogenesis by VPA is also closely linked with the epigenetic mechanisms deriving from HDAC inhibition [130]. In detail, VPA significantly inhibited seizure-induced neurogenesis and potently protected epilepsy-associated cognitive deficit in rodent model after KA-induced seizures via inhibition of HDACs. The epigenetic inhibition of VPA is also involved in the regulation of synaptic plasticity via BDNF/TrkB pathway. This pathway is critical for induction and preservation of striatal plasticity in a genetic mouse model of epilepsy which is associated with the Bassoon gene (Bsn) mutation [136, 137]. Pharmacological inhibition of TrkB by subchronic intrastriatal administration of k252a rescued behavioral alterations in Bsn mutant mice and prevented the occurrence of pathological form of plasticity in fast-spiking interneurons. Thus, the direct modulation of VPA on synaptic plasticity can be achieved by seizure reduction and epigenetic mechanisms. In a mouse model with heterozygous mutants of tuberous sclerosis complex 2 (TSC2), which is a cause of tuberous sclerosis manifesting with seizures, autism, and cognitive deficits [138], a global reduction of histone H3 acetylation was found in hippocampus tissues. Pharmacological inhibition of HDAC by VPA restored histone H3 acetylation levels, reversed synaptic plasticity aberrations and improved the epileptic phenotype in TSC2^+/−^ mice. Due to the use of the pan HDACi in the present work, the detailed HDAC isoform has not been definitively identified for the contribution to the neurological manifestations in the TSC. It is likely that genetic silencing of each HDAC isoform is required to determine which type of HDAC is vital for TSC. Although there are multiple of other targets such as inactivation of sodium channel [139] and enhancement of γ-aminobutyric acid (GABA)-mediated inhibitory neurotransmission [140], pan HDACs inhibition is believed to play a critical role for the therapeutic value of VPA as mentioned above.

**Table 2:** HDACi reported in the treatment of epilepsy rodent models

HDACi	Target	Disease model	Species	Results	Refs
VPA	HDAC I & II	PTZ-induced SE KA-induced SE Genetic model of absence epilepsy	Mouse Mouse Rat	Increase of seizure threshold Decreases of hippocampal neurogenesis and cognitive deficits Increase of histone acetylation and inhibition of epileptogenesis	[141] [130] [142]
TSA	HDAC I & II	Pilo-induced SE KA-induced SE KA-induced SE	Mouse Mouse Rat	Increase of GluR2 expression Increase of histone hyperacetylation Increase of GluR2 expression	[143] [144] [130]
NaB	HDAC I & II	ES Kindling model Genetic model of absence epilepsy	Mouse Mouse Rat	Increases of H3 and H4 acetylation Improving anticonvulsant activity of MK-801 Preventing mossy fiber sprouting in hippocampus; Promoting acetylation of H3 and H4 histones; Inhibiting epileptogenesis	[145] [132]

*Note* VPA, valproic acid; PTZ, pentylenetetrazole; KA, kainic acid; Pilo, pilocarpine; TSA, trichostatin A; GluR2, metabotropic glutamate receptor 2; ES, Electroconvulsive seizure; NaB, Sodium butyrate

Sodium butyrate (NaB), a natural SCFA exhibiting a wide spectrum of inhibition of HDACs, has been reported to possess pronounced anti-epileptic effect in several mouse models. In a rat hippocampal kindling model, NaB was found to significantly inhibit total HDAC activity and display an evident reduction of mossy fiber sprouting, an index of epileptogenesis [146]. Administration of NaB enhanced the acetylation level especially H4 histone in the mouse hippocampus and cerebral cortex, accompanied with the elevation of IEGs (c-fos and Arc) and neurotrophins (BDNF and NT-4) [147]. In the WAG/Rij epilepsy rat model, a genetic model of absence epilepsy, epileptogenesis, and mild-depression comorbidity, the acetylated levels of H3 and H4 histones were increased significantly and the decreased expressions of HDAC1 and HDAC3 were observed following NaB treatment [132]. It suggests that inhibition of HDAC by NaB might be a powerful approach for preventing the development of epileptogenesis also affecting behavioral comorbidities. In the stress state by forced swimming in cold water, NaB could restore the anti-seizure efficacy of MK801, a non-competitive NMDA receptor antagonist, via increasing the acetylation status of H3 and H4 histone proteins and promoting gene expression [145]. Epigenetic intervention by NaB had the capacity to delay appearance of epilepsy and reduce seizure severity. There was also evidence supporting a persistent effect of NaB on functional improvement in the hippocampus kindling model of temporal lobe epilepsy [146]. Altogether, these findings envisage a promising therapeutic potential of NaB against epilepsy.

Trichostatin A (TSA) is another promising HDACi in the family of hydroxamate. It was previously reported that TSA could restore the deacetylation of H4 histone and increase the transcriptional level of glutamate receptor 2 (GluR2) in the hippocampal CA3 subregion in a rat model of pilocarpine-induced SE, thus attenuating seizure-induced neuronal injury [143]. There is prior work demonstrating that the rapid induction of gene-specific alterations in histone acetylation patterns serves as an early step in the pathological processes caused by SE. In detail, it has been demonstrated that acetylation of histone H4 was diminished in rat hippocampus was reduced at the glutamate receptor 2 (GluR2) promoter after SE and H4 deacetylation appeared prior to GluR2 mRNA downregulation. Additionally, TSA could quickly prevent deacetylation of GluR2-associated histones and suppressed the transcriptional level of GluR2 post seizures [143]. Therapeutic effect of TSA was also observed in a KA-induced acute seizure model, since TSA evidently enhanced the acetylated levels of H3 and H4 histones at the Nrg1 promoter locus and subsequently resulted in the increase of neuregulin 1 expression [148]. These studies suggest that TSA functions as an effective epigenetic agent in seizure suppression and associated consequences. HDAC inhibition by TSA can modify epileptogeneic process following SE. Further study is essential to clarify this item.

Vorinostat (SAHA) is another broad-spectrum HDACi subordinated to the hydroxymate family with the inhibitions of class I, II and IV HDACs. It has been widely accepted for clinical treatment of cancers. Recently, this agent has yielded encouraging evidence showing beneficial effects in epilepsy models. In the rat seizure model induced by KA, pretreatment with SAHA obviously augmented seizure latency and diminished seizure severity [149]. It also suppressed neuronal apoptosis and the microglial activation. Mechanistically, SAHA pretreatment resulted in the decrease of histone H3 acetylated at lysine 9 (H3K9) of TLR4 gene and inhibited TLR4/MyD88 signaling pathway, finally protected from KA-induced brain damage [150]. The inhibitory effects of HDAC1 and HDAC10 expressions were also observed in the zebrafish epilepsy model triggered by a GABA_A_Rγ 2 subunit (GABRG2) mutation after treatment with SAHA [151]. More importantly, SAHA exhibited the shorter onset time and more satisfactory therapeutic efficacy than the conventional AEDs in this model. In addition, pharmacological intervention with SAHA also restored the histone H3 acetylation level, reversed pathological form of synaptic plasticity, and abrogated the epileptic phenotype in the TSC2^+/−^ mice showing the neurological manifestations including seizures, autism and cognitive deficits [138]. These data suggest that SAHA may act as a potent neuroprotective and anticonvulsant agent via epigenetic regulation.

So far, pharmacological intervention is a prevalent strategy for exploring the role of HDACs in regulating histones and non-histones in epilepsy. However, the vast majority of HDACi exhibit global inhibition of diverse HDACs isoforms. In this condition, the mice deficiency of the particular subunit could be a powerful tool to analyze the involvement of HDACs in the occurrence of seizure and development of epileptogenesis. Besides, in epileptic condition, there are scarce information on the effects of HDACi on the cellular types and brain regions. However, as these aspects have been demonstrated to affect neural circuits and key events involving in the etiology of epilepsy [152], it requires further exploration for precise manipulation of epilepsy treatment in the future.

### Applications of HDACi in the treatment of patients with epilepsy

The most successful example supporting the application of HDACi for the clinical treatment of epilepsy is VPA, which has been approved for counteracting generalized and focal seizures since 1970s (Table 3). High efficacy of VPA is verified in patients diagnosed as typical and atypical absence seizures both in childhood and adulthood [153]. Due to the accurate validation of the clinical efficacy in both randomized controlled trials and observational studies [154, 155], VPA acts as a mainstay of therapy for almost all seizure types in patients with all age group. In the randomized controlled trial carried out by Glauser and colleagues, it was found that initial monotherapy with VPA for children with newly diagnosed childhood absence epilepsy exerted superior efficacy for seizure control without unsatisfactory adverse events compared to other AED such as lamotrigine [156]. Recently, an updated Cochrane review was carried out to summarize the evidences regarding the therapeutic effects of ethosuximide, VPA and lamotrigine on absence seizures in children and adolescents [157]. It was found that VPA was the preferable choice for the treatment of patients with coexistence of absence and generalised tonic-clonic seizures. In terms of patients diagnosed with idiopathic generalized epilepsy or unclassifiable seizures, VPA remains also the candidate while lamotrigine and topiramate should not be considered in these patients due to lower efficacy and intolerable profiles [158]. In addition, available evidences also support VPA efficacy in a variety of epilepsy syndromes [159] including Lennox-Gastaut syndrome (dosage ranged from 500 mg/day to 1500 mg/day [160]), West syndrome (dosage at 30 mg/kg/day [161]) and Dravet syndrome (dosage ranged from 25 to a maximum of 60 mg/kg/day [162]). Finally, it is important to mention that the beneficial effect of VPA on women of childbearing years should be reassessed as a result of teratogenicity reported during pregnancy [163]. In the last decades, VPA also has garnered much interest in the treatment of several conditions other than epilepsy. It has been illustrated that VPA is approved by the U.S. Food and Drug Administration and the European Medicines Agency (FDA/EMA) for curing patients with bipolar disorders or migraine prophylaxis. There are also potential therapeutic implications in Alzheimer’s disease and in cancer therapy [133]. The multiple therapeutic paradigms of VPA may be attributable to various mechanisms of action (MoAs), especially epigenetic effects. In epilepsy research, although there are several reports illustrating that enhancement of GABA-mediated inhibitory neurotransmission [139] and ion channel inhibition [140] are involved in the convulsive effects of VPA, there is also prior work supporting that HDACs serve as vital upstream regulators of molecular targets associated MoAs such as ion channel [164]. We believe that HDACs may be critical factors for initiating MoAs to explain the anti-convulsive effects of VPA. Further investigation is indispensible to clarify this item.

**Table 3:** Summarization of HDACi in clinical studies

Agent	Disease	Identifier	Status
VPA	Epilepsy	Clinical drug	Clinical drug
LTG	Epilepsy	Clinical drug	Clinical drug
LCM	Epilepsy	Clinical drug	Clinical drug
SAHA	Hodgkin lymphoma	Clinical drug	Clinical drug
SAHA	NSCLC	Clinical drug	Clinical drug
SAHA	Ovarian cancer	Clinical drug	Clinical drug
Chidamide	PTCL	Clinical drug	Clinical drug
VPA	Schizophrenia	NCT00194025	Phase IV
VPA	AD	NCT00071721	Phase III
VPA	Bipolar disorder	NCT00431522	Phase IV
VPA	Spinal muscular atrophy	NCT00481013	Phase II
VPA	Acute cerebral hemorrhage	NCT01115959	Phase IV
VPA	Retinitis pigmentosa	NCT01233609	Phase II
DVPX	Autism	NCT00211757	Phase II
DVPX	Mood disorder	NCT00217932	Phase II
SAHA	Aerodigestive tract tumors	NCT00735826	Completed
SAHA	Breast cancer	NCT00719875	Phase I
SAHA	AML	NCT00305773	Phase II
SAHA	Multiple myeloma	NCT00773747	Phase III
SAHA	Kidney cancer	NCT00278395	Phase II
SAHA	Liver cancer	NCT01075113	Phase I
Romidepsin	HIV	NCT01933594	Phase I/ II
Romidepsin	Colorectal cancer	NCT00077337	Phase II
Entinostat	Breast cancer	NCT02833155	Phase I
Phenyl butyrate	MSUD	NCT01529060	Phase II/ III
Belinostat	PTCL	NCT00865969	Phase II
Belinostat	Solid tumor; Lymphoma	NCT00413075	Phase I
Belinostat	Neoplasms; Lymphoma	NCT01273155	Phase I
Belinostat	Thymoma; Thymic carcinoma	NCT00589290	Phase II
Quisinostat	Lymphoma; Neoplasms	NCT00677105	Phase I
Quisinostat	Ovarian cancer	NCT02948075	Phase II

*Note* VPA, valproic acid; LTG, lamotrigine; LCM, lacosamide; SAHA, suberoylanilide hydroxamic acid; NSCLC, non-small cell lung cancer; PTCL, peripheral T-cell lymphoma; DVPX, divalproex sodium; AD, Alzheimer’s disease; HDAC, histone deacetylase; AML, acute myeloid leukemia; MSUD, maple syrup urine disease; HIV, human immunodeficiency virus

Lacosamide (LCM), a novel drug with the reduction of HDAC1 protein expression in preclinical studies as mentioned above, was licensed in 2008 by FDA/EMA as an adjunctive treatment for curing partial-onset seizures in adolescent patients attacked by epilepsy. The first large clinical trial was carried out David Rudd research group as an international, multicenter trial with three treatment schemes (200, 400, and 600 mg/day) [165]. The major results demonstrated that adjunctive LCM exhibited a dose-dependent increase in therapeutic efficacy and decrease in retention rate. LCM at the dose of 400 mg/day seems to be the most satisfactory tradeoff in the aspects of efficacy and tolerability. In the second RCT initiated by the SP755 Study Group, LCM was treated with two schemes including 200 and 400 mg/day [166]. It was found that treatment of LCM with two dose regimes both resulted in the reductions of median seizure frequency. The third clinical trial launched by the SP754 Study Group which solely recruited American patients also confirmed the long-term efficacy and tolerability of adjunctive LCM in the treatment of epilepsy [167]. Recently, in multicenter real-world study in China, the add-on treatment with LCM for children and adult focal epilepsy led to the evident therapeutic efficacy with favorable safety [168, 169]. Despite appearance of some adverse effects including dizziness, headache and nausea, these events are generally mild and well tolerated. LCM represents a reliable agent for the recommendation as adjunctive therapy of partial-onset seizures in adults. Further research is warranted to expand its field of use in the treatment of epilepsy.

## Concluding remarks and perspectives

Epilepsy has become a public threat throughout the world. Current medications provide symptom relief rather than exerting disease-modifying effect. In addition, despite great progress regarding the role of nonsense-mediated decay of SCN1A in disease modification [170, 171], there is still no approach to modify epilepsy in clinical practice. In the above article, we describe the updated roles of HDACs in the pathophysiology of epilepsy and the therapeutic effects of diverse HDACi, it is plausible that the epigenetic targets may attract much attention in the development of disease-modifying drugs for dealing with epilepsy. To our knowledge, 18 HDACs in humans may have distinct roles in epilepsy. Class I HDACs appear to be neuroprotective while class II HDACs likely have neurotoxic effects. It is particular to note that specific blockade of HDAC1 protects against diverse neurological condition including epilepsy. In contrast, activation of SIRT1, a member of class III HDACs, always reduces the epilepsy progression. Therefore, the modulation of HDACs may serve as an intriguing therapeutic approach for curing epilepsy.

Despite some studies of various HDACs in rodent epilepsy models and clinical patients, the detailed regulatory mechanism in epilepsy is still very limited. Genetic silencing or isoform-specific inhibitor of HDAC is required in the future to clarify the exact role of each HDAC isotype following epileptic condition. Nowadays, although some HDACi including VPA, lamotrigine and SAHA [172] are approved or enrolled in clinical trials for the treatment of epilepsy, they always show some adverse effects such as hepatic injury, teratogenicity and so on, which are largely attributed to the global inhibition of HDACs for these drugs. In addition, the vast majority of current available HDACi are hydrophilic substances and the blood-brain barrier (BBB) prevents these compounds from entering the brain to exert therapeutic potential. On the one hand, BBB penetration can be improved through the structural modification of small molecules [173], finally increasing brain exposure. On the other hand, the emerging proteolysis targeting chimera (PROTAC) technology, which was introduced by Crews and Deshaies in 2001 via disrupting the endogenous ubiquitin (Ub)-E2 ligase-E3 ligase complex [174], which is widely applicable for degrading abnormal protein targets including epigenetic proteins, may show promise in the treatment of epilepsy. In principle, there are three structural units for PROTAC, which includes the E3 ligase binding motif for the recruitment of the ubiquitin-proteasome-system (UPS), a linker and a second pharmacophore for the binding of the target protein [175]. The simultaneous interaction with the endogenous Ub-E2-E3 complex and the protein of interest results in the generation of an Ub-E2-E3-neosubstrate complex, ultimately leading to ubiquitinylation and degradation of target protein. Unlike the conventional small molecule inhibitors, the PROTAC technology possesses its unique catalytic mode of action. Since one PROTAC molecule can proceed multiple degradation cycles, it usually requires substoichiometric engagement of target protein. Besides, PROTAC often exerts its function via event-driven mode of action rather than occupancy-driven mode of action of traditional small molecule inhibitors, which suggests that this technology does not require high-affinity binding. With this in mind, the resistance of small molecule agonists or antagonists appears due to reduction of the binding affinity by mutation of target, PROTAC is likely to overcome this challenge. Furthermore, development of small molecule agonists or antagonists is often performed via binding a certain and definite site in the target molecule while PROTAC is not dependent on these certain sites as it is implemented via the efficient building of a ternary complex between the PROTAC molecule, the protein of interest and the E3 ligase. In comparison to HDACi, PROTAC has diverse advantageous properties notably development of selective HDAC isozyme inhibitor and prevention of drug resistance. Currently, the approved HDACi in clinical practice has limited application possibly due to the pan-HDAC inhibition profile. In this point, some HDAC-PROTACs have been demonstrated to selectively degrade HDAC isoform for disease treatment. For instance, selective degradation of HDAC6, HDAC8 and SIRT2 by PROTACs has been reported to inhibit tumor progression in vitro [176–178]. Despite no report on the application of PROTAC via targeting HDAC isoform in the field of neuroscience until now, there are publications supporting that PROTAC can be employed to degrade other target protein for improvement of brain disease. For example, tau, a pathological hallmark of Alzheimer’s disease, was observed to successfully degraded in human neurons [179]. In the field of Huntington’s disease, there are also small-molecule PROTACs that are effective for degrading huntingtin in patient-derived fibroblasts [179]. These studies have the prospect of treating neurodegerative diseases [180], we believe that PROTAC holds promise in the treatment of neurological conditions including epilepsy via selectively targeting HDAC isoform in the future.

## Data Availability

No datasets were generated or analysed during the current study.

## Abbreviations

CNS: Central Nervous System
EEG: Electroencephalogram
ILAE: International League Against Epilepsy
DRE: Drug-Resistant Epilepsy
AEDs: Anti-Epileptic Drugs
HDAC: Histone Deacetylase
HDACi: Inhibitors of HDACs
DVPX: Divalproex Sodium
SIRT: Sirtuin
NAD^+^: Nicotinamide Adenine Dinucleotide
MAP: Mitogen-Activated Protein
MKP1: MAP Kinase Phosphatase 1
MAPK: MAP Kinase
SMRT: Silencing Mediator for Retinoid and Thyroid Receptors
N- CoR: O- Nuclear Receptor Corepressor
LPS: Lipopolysaccharide
STAT1: Signal Transduction and Activation Of Transcription 1
NF-κB: Nuclear Factor κB
MDM2: Murine Double Minute 2
ISG: IFN-Stimulated Gene
MEF2: Myocyte Enhancer Factor-2
Hsp90: Heat Shock Protein 90
SUMO: Small Ubiquitin-Like Modifier
ZXD: Zinc finger X-linked Duplicated
ZXDC: ZXD Family Zinc Finger C
PML: Promyelocytic Leukemia Protein
SCFA: Short Chain Fatty Acid
IEG: Immediate Early Gene
BDNF: Brain-Derived Neurotrophic Factor
PP2A: Phosphatase 2 A
RGC: Retinal Ganglion Cell
GABA: Gamma-Aminobutyric Acid
SRF: Serum Response Factor
HS: Hippocampal Sclerosis
MTLE-HS: Mesial Temporal Lobe Epilepsy with Hippocampal Sclerosis
PGC-1α: Proliferator-Activated Receptors and γ coactivator 1α
VPA: Valproic Acid
LCM: Lacosamide
Bsn: Bassoon Gene
TSC2: Tuberous Sclerosis Complex 2
NaB: Sodium Butyrate
TSA: Trichostatin A
GluR2: Glutamate Receptor 2
SAHA: Vorinostat
GABRG2: A Type GABA receptor γ 2
FDA: Food and Drug Administration
EMA: European Medicines Agency
BBB: Blood-Brain Barrier
PROTAC: Proteolysis Targeting Chimera
